# Factors Associated with Antiretroviral Therapy Toxicity Out-Comes in Patients with and without Hypertension

**DOI:** 10.3390/ijerph191711051

**Published:** 2022-09-03

**Authors:** Sabelo Bonginkosi Dlamini, Ming-Tsang Wu, Hans-Uwe Dahms

**Affiliations:** 1 Department of Public Health, Kaohsiung Medical University, Kaohsiung 80708, Taiwan; 2 PhD Program in Environmental and Occupational Medicine, Kaohsiung Medical University, Kaohsiung 80708, Taiwan; 3 Research Center for Precision Environmental Medicine, Kaohsiung Medical University, Kaohsiung 80708, Taiwan; 4 Department of Family Medicine, Kaohsiung Medical University Hospital, Kaohsiung Medical University, Kaohsiung 80708, Taiwan; 5 Department of Biomedical Science and Environmental Biology, Kaohsiung Medical University, Kaohsiung 80708, Taiwan; 6 Department of Marine Biotechnology and Resources, National Sun Yat-sen University, Kaohsiung 80708, Taiwan

**Keywords:** hypertension, antiretroviral therapy toxicity, estimated glomerular filtration, alanine aminotransferase, aspartate aminotransferase

## Abstract

Background: Negative effects of antiretroviral therapy (ART) drugs on HIV/AIDS patients are one of the major health issues in the therapeutic treatment of this communicable disease. This holds particularly for people living with HIV (PLHIV) who might have a non-communicable disease (like hypertension), which also requires a lifetime treatment. In this study, we investigated the association between hypertension and other possible factors on ART toxicity markers in patients with hypertension, compared to those without hypertension. Methods: This retrospective longitudinal study reviewed chronic patient files of 525 patients (of which 222 were hypertensive) who satisfied the inclusion criteria and were on ART at a hospital in central Eswatini. Specific levels of estimated glomerular filtration (eGFR), alanine aminotransferase (ALT) and aspartate aminotransferase (AST) were used as drug toxicity markers. To analyze the longitudinal data between the exposure of interest and outcome variables, a Generalized Estimated Equation method was employed. Results: Participants with hypertension had decreased eGFR compared to those without hypertension (β = −2.22; *p*-value = 0.03). There was no significant association between ALT, AST and hypertension (*p*-value = 0.34 and 0.20, respectively). Factors that were found to have a significant association with ART toxicity markers included age, sex, ART duration, hypertension treatment and time of study. The eGFR was found to be significantly increasing over the study period (*p*-value < 0.001) for all participants. The significance was consistent in both hypertensive and non-hypertensive participants independently (*p*-value = 0.002 and <0.001, respectively). The overall trends of ALT and AST over time were also significant (*p*-value = 0.003 and <0.001, respectively). Conclusions: Patients with hypertension had decreased eGFR, and there was a significant association of eGFR with time of the study. Special attention, therefore, to monitor calamities which are indicated by a decrease of eGFR (like renal impairment) should be given in PLHIV on ART with hypertension, especially more so if they were on ART for longer time.

## 1. Introduction

Sub-Saharan Africa is experiencing an emerging burden of non-communicable diseases (NCDs), more especially because certain risk factors of NCDs are also becoming more popular as lifestyle change and rates of urbanization increase [1]. Most NCDs are chronic. Chronic diseases are conditions requiring an ongoing medical treatment and they have an impact on a person’s daily life [2]. Hypertension is one of the most common NCDs globally, and it serves as a primary disposing factor for other well-known NCDs [3]. For that reason, hypertension is used in this study as a proxy or marker for NCDs. In treating hypertension, different classes of drugs (like diuretics and beta-blockers among others) are used to lessen high blood pressure [4].

As the introduction of antiretroviral therapy (ART) has prolonged the lives of people living with HIV (PLHIV), there is an increasing likelihood for them to also have NCDs requiring lifetime treatment. In fact, there is evidence that the burden of NCDs like cardiovascular conditions is increasing among PLHIV compared to the general population [5]. A large-scale study of 5563 patients on ART in Uganda found that 27.9% of the patients were diagnosed with hypertension [6]. In another study conducted in Cameroon, results revealed that the prevalence of hypertension in patients on ART was twice that of ART-naïve patients [7]. Most of these studies between ART and hypertension focused on hypertension occurring as a result of the initiation of HIV treatment. However, our present investigation was based on hypertension being independent from HIV or ART since our study population had hypertension before the initiation of ART. Despite substantial evidence that has documented the benefits of ART, it is, however, less known whether the co-existence of NCDs and HIV will affect the progress of each other during their respective treatments.

One of the fears of PLHIV is experiencing vast side effects of the ART treatment. That on itself can have so much effect on influencing the patients’ willingness to start treatment, and whether to adhere and comply with the treatment when they have finally decided to start it. The WHO and the U.S. Department of Health of Human Services stated the following possible side and adverse effects of ART: drug-induced bone-marrow suppression, lactic acidosis, hepatic toxicity, pancreatitis, and peripheral neuropathy, fat maldistribution and body habitus changes, hyperlipidemia, hyperglycemia, insulin resistance, diabetes mellitus, osteopenia, osteoporosis and osteonecrosis, skin rashes and hypersensitivity reactions, nausea, diarrhea, dizziness, suicidal ideation, abnormal dreams, depression, myocardial infarction, ischemic stroke, proximal renal tubulopathy and elevated creatinine, nephrolithiasis, and cholelithiasis [8,9].

As liver toxicity (hepatotoxicity) is one of the common toxicities caused by ART among PLHIV, the contribution of each particular anti-HIV drug to the development of hepatotoxicity in ART is difficult to determine. There are multiple possible pathogenic mechanisms that can be involved in hepatotoxicity, which can include direct drug toxicity, immune reconstitution in the presence of Hepatitis C virus and/or Hepatitis B virus co-infections, hypersensitivity reactions with liver involvement, and mitochondrial toxicity. Other pathogenic pathways that may be involved can include insulin resistance caused by several anti-HIV drugs, which may facilitate the development of steatohepatitis [10]. Liver damage or injury can be suspected when abnormalities are seen with liver tests. There is a broad variability among studies in the criteria to categorize the severity of hepatotoxicity. However, we propose the AIDS Clinical Trials Group scale of liver toxicity, for this study. According to it, patients with transaminases within normal limits at baseline are considered to develop hepatotoxicity when alanine aminotransferase test (ALT) and/or aspartate aminotransferase test (AST) rise above the upper limits of normal range with time. Severe hepatic injury is defined as a grade 3 or 4 change in AST and/or ALT levels during ART. If AST and ALT grades were discordant, the highest values should be used for classification purposes [11].

As ART is a life-time treatment, there is an increasing need for monitoring toxicity of anti-HIV drugs. According to the WHO, monitoring of ART toxicity is important also for particular groups, which include those using the treatment during pregnancy and breastfeeding, in infants and children, and by people who use them mainly to prevent HIV infection, such as in sero-discordant couples [12]. In Eswatini, the routine check-up of liver function (by means of ALT and AST) and renal function (by means of serum creatinine) is one of the criteria used to monitor ART toxicity regularly. Hence in our study it has been the convenient criteria for determining the liver function, as these tests are readily available. On a different note, it is vital to understand that LFTs might not be directly correlated with liver problems but could be contributed by other factors. However, they are a good start for further investigations when it comes to liver function, more especially in patients on ART. For that reason, we use more than one liver function test, and repeated measures in our study to minimize this kind of limitation.

As it became evident that PLHIV who were on ART do experience a considerable amount of side effects due to the treatment, such reasons and the fact that clients who were on ART had to take the treatment for the rest of their lives can precipitate poor progress with the treatment due to the likelihood of non-adherence and non-compliance to treatment. If the burden can be this heavy with the PLHIV who are on ART, one then can imagine how much more the burden can be if they also have a chronic NCD which also requires a consistent life-time treatment. Such an instance is likely to even aggravate the level or chances of drug toxicity. In this study we, therefore, investigated the association of hypertension on ART toxicity biomarkers (liver and kidney function tests) and determined possible confounding factors associated with ART toxicity markers in patients with hypertension and those without hypertension.

## 2. Materials and Methods

### 2.1. Study Design and Sample

This is a retrospective longitudinal study using the available patient records and files from the Raleigh Fitkin Memorial (RFM) Hospital in Eswatini. This is one of the largest healthcare facilities right in the center of the country. The target population were all patients who were on ART and with an NCD in Eswatini. The source and study population were patients on ART and with hypertension in the RFM Hospital, who were monitored under the ART department. The health facility in this study was purposely sampled, more so because it was making effort to integrate HIV/AIDS mitigation services and NCD services which increase the chances of acquiring subjects with co-existing HIV/AIDS and hypertension.

Patients included in this study were selected based on the following criteria: 30 years or older; for 2 years or more on ART; and having normal liver and renal function before the initiation on ART. Lastly, for the patients with hypertension, they had to be diagnosed with hypertension before ART initiation in order to rule out hypertension that possibly resulted from ART which can bias the results. If patients had other NCDs or other communicable disease(s), using hormonal contraceptives, and receiving treatment of any other illness besides HIV/AIDS and hypertension, their files were excluded from this study. This was done because these variables were likely to act as confounding factors and then directly affect the prognosis of HIV/AIDS and hypertension. With reference to the sample size, only 525 patients (303 without hypertension and 222 with hypertension) were selected for in-depth reviews as they met the inclusion criteria and had almost all information needed for this study.

### 2.2. Dependent and Independent Variables

For this study, the dependent variables were ART toxicity markers. To determine the ART toxicity markers, liver and renal function laboratory tests were used. For liver function, the AST and ALT were used, as the laboratory tests which are commonly used in Eswatini hospitals for PLHIV to determine their liver function. For renal function, the serum creatinine (SCr) test was collected as it is the commonly used test to assess renal function in PLHIV in Eswatini, and for this study the SCr (together with age, race, and sex) was used to calculate eGFR, which was one of the dependent variables for determining ART toxicity with regard to renal function. The threshold for AST and ALT according to McAuley and Park et al., is 35 U/L [13,14]. For a desirable outcome in this study, the AST and ALT had to be decreasing. With regard to eGFR, normal figures have to be >90 mL/min/1.73 m^2^. In calculation of the eGFR, the CKD-EPI creatinine equation was used (eGFR = 141 × min (S_Cr_/κ, 1)^α^ × max (S_Cr_ /κ, 1)^−1.209^ × 0.993^Age^ × 1.018 [if female] × 1.159 [if Black]) [15]. This equation was chosen over the others (like the MDRD study equation) because of its accuracy when eGFR was >90 mL/min/1.73 m^2^ [16] and its precision in diverse populations [17]. Therefore, for desirable results in this study, the eGFR had to be increasing.

The presence of hypertension was deemed as the main independent variable for this study. Other independent variables chosen based on the literature included ART Regimen, ART duration, ART substitution, adherence, age, sex, isoniazid preventive therapy (IPT), settlement, WHO HIV staging, year of study, hypertension treatment, and adverse effects to ART. The adherence was categorized into three levels as recorded on the chronic patient files: poor, good, and excessive. The pill count (when patients come for medication refill) is basically the way used to calculate adherence in the hospital. Pill count less than 90% indicated poor adherence, 95–105% indicated good adherence, and >105% indicated excessive adherence or overuse, which might be an indication of overdose or the fact that the patient might be sharing the pills with someone else. The treatment of hypertension was categorized into lifestyle modifications, single-drug class, two-drug classes and three-drug classes. For a single-drug class, those are participants who were on either one of the following: diuretics, ACE inhibitor or calcium channel blockers. For two-drug classes the participants were in any combination of two of the mentioned drug classes, with beta blockers in consideration. Lastly, those on three-drug classes were in combination of three of the already mentioned classes of drugs. On the other hand, lifestyle modification had to do with healthy habits such as regular exercise, low fat and low salt diet.

### 2.3. Data Collection and Management

A 28-item case-report form was the instrument used for data collection, having chronic patient files as the sole data source. It had three sections: one collecting socio-demographic data, another collecting medical history, and a third collecting laboratory results. The data were collected from 27 July 2018 to 8 September 2018 by the principal investigator. The file review was in two stages: the quick review (which involved all the chronic patient files in the ART department) and in-depth review (which involved patient files that during a quick screening met the inclusion criteria).

### 2.4. Data Analysis

An SPSS software version 28 (by International Business Machines Corporation, Armonk, NY, USA) was used for running the analysis in this study. Chi-square (χ²) test was used to compare proportional differences between different levels of categorical independent variables. A *p*-value < 0.05 was considered as statistically significant. For multivariable analysis, the SCr, AST, and ALT were recorded as continuous variables and with repeated measurements (for 2015, 2016, 2017 and 2018). Therefore, a generalized estimation equations (GEE) model was employed to study the relationship between the independent variables and the dependent variables in a whole-group analysis and subgroup analysis. Beta coefficients with *p*-values were reported.

## 3. Results

### 3.1. Demographic and Clinical Characteristics of Participants

Based on Table 1, the mean age of participants that were involved in the study was 44.25 ± 8.66 (41.99 ± 7.14 for non-hypertensive participants and 47.34 ± 9.56 for hypertensive participants). Participants with the age of 30–40 years of age (43.6%) were the majority, followed by those with age of 41–50 years (33.9%), then lastly those with >50 years (22.5%). With regard to the presence of hypertension, 54.1% of participants without hypertension were 30–40 years, yet with participants having hypertension the majority were of 41–50 years of age (35.6%). With regard to sex, the majority of the participants were females (62.1%), and such proportion was true for both groups (hypertensive and non-hypertensive) of participants independently.

Regarding the participants’ clinical characteristics, the majority were on stage 1 of the W.H.O. HIV staging (94.7%), which also applied for both groups of participants independently, with a statistically significant difference (*p*-value = 0.01). With regard to ART duration, the participants’ mean duration on ART was 5.53 ± 1.52. Majority of the participants were on EFV-based therapy (65.5%), followed by those who were on NVP-based therapy (32.5%). Such proportion applied for both groups of participants, whereby 55.4% of the participants with hypertension were of EFV-based therapy (which is the majority) and 72.9% of such had no hypertension. About 3.4% of the participants had ART adverse effects (non-hypertensive 2% and hypertensive 5.4%) and such difference between the groups was statistically significant (*p*-value = 0.03).

With regard to hypertension treatment, most of the hypertensive participants were on a single drug class (36%). That is to say they were either on diuretics, ACE inhibitors or calcium channel blockers. The following group was on lifestyle modifications (31.5%). Those who were taking a combination of three drugs classes were the least (7.7%). The difference between all the hypertension treatment levels was significant (*p*-value < 0.001).

### 3.2. ART Toxicity Outcomes

Table 2 summarizes the three ART toxicity outcomes used in this study. Participants without hypertension had higher eGFR means throughout the course of the study compared to participants with hypertension. Considering all the participants, the eGFR means were slightly increasing as time went by (from 102.52 ± 24.29 in 2015 to 109.28 ± 23.86 in 2018). With regard to ALT, the means were demonstrating a U-shaped pattern as time went by (31.77 ± 35.27 [2015], 29.97 ± 22.94 [2016], 28.99 ± 16.96 [2017], and 30.67 ± 32.02 [2018]). The U-shaped pattern observed in ALT and AST was also consistent in both groups of participants independently.

### 3.3. Association between Independent and Dependent Variables in All Participants (Whole-Group Analysis)

According to the whole-group analysis in Table 3, participants with hypertension had a significantly decreased eGFR compared to participants with hypertension after adjustment of the other variables (β = −2.22; *p*-value = 0.03). With regard to age, participants >50 years and 41–50 years had a significantly decreased eGFR compared to participants who were 30–40 years old (β = 20.29; *p*-value < 0.001 and β = −8.96; *p*-value < 0.001, respectively). Another factor that was found to have a significant association with eGFR was sex. Males had increased eGFR compared to females after adjustment of the other variables (β = 3.94; *p*-value = 0.02). ART duration also had a significant association with eGFR after adjustment of the other variables. With each and every 5 years increase on ART duration, the eGFR was decreasing (β = −1.37; *p*-value = 0.02). With regard to hypertension treatment, participants who were taking two drug classes had decreased eGFR compared to participants who were on lifestyle modification (β = −7.86; *p*-value = 0.03). Regarding participants who were on a single drug class or taking three drug classes, the association was not significant (*p*-value = 0.14 and *p*-value = 0.35, respectively). Factors like ART regimen, W.H.O. HIV Stage, ART adherence and settlement did not have any significant association with eGFR in the whole-group analysis.

With regard to liver function outcomes (ALT and AST), as it can be seen in Table 4 and Table 5, age and sex were the only factors significantly associated with ALT in the whole-group analysis. There were no factors found to be significantly associated with AST. According to Table 4, participants with hypertension had increased ALT compared to participants without hypertension, after adjusting for the other variables. However, such association was not significant (β = 2.55; *p*-value = 0.34). The Table 4 results also reveal that males had increased ALT compared to females after adjustment of other variables (β = 3.44; *p*-value = 0.01).

In Table 5, hypertension was also not significantly associated with AST. Participants with hypertension had decreased AST compared to participants without hypertension after adjusting for the other variables (β = −2.11; *p*-value = 0.20).

### 3.4. Association between Independent and Dependent Variables in Non-Hypertensive and Hypertensive Participants Independently (Sub-Group Analysis)

According to Table 3, the sub-group analysis revealed that age (just like in the whole-group analysis) was significantly associated with eGFR, in both hypertensive and non-hypertensive participants, independently. In non-hypertensive participants, those who were 41–50 years old and >50 years old had decreased eGFR compared to participants who were 30–40 years old (β = −8.47; *p*-value < 0.001 and β = −21.94; *p*-value < 0.001, respectively). Such association was also consistent in hypertensive participants (β = −8.82; *p*-value = 0.004 for age group 41–50 years and β = −19.12; *p*-value < 0.001 for age group > 50 years). Sex was found to be significantly associated with eGFR in participants with hypertension in the sub-group analysis whereby males had increased eGFR compared to females after adjustment of the other variables (β = 6.98; *p*-value = 0.01). With regard to hypertension treatment, participants who were taking combination of two drugs classes had decreased eGFR compared to participants who were on lifestyle modification (β = −7.53; *p*-value = 0.04). ART duration was found to be significantly associated with eGFR in participants without hypertension (β = −1.60; *p*-value = 0.04).

Based on Table 4 sub-group analysis, sex was the only factor found to be significantly associated with ALT in the sub-group analysis for both hypertensive and non-hypertensive participants independently (β = 7.04; *p*-value = 0.01 and β = 12.23; *p*-value < 0.001, respectively). With regard to AST, in Table 5 sub-group analysis, ART duration was significantly associated with AST in participants without hypertension (β = −1.13; *p*-value = 0.01). In participants with hypertension, ART duration did not have a significant association with AST (β = 0.53; *p*-value = 0.22).

### 3.5. Overall Trends of eGFR, ALT and AST with and without Hypertension

As it is evident in Table 3, the eGFR was increasing as time went by, comparing the year 2015 to the subsequent years of the study (2016, 2017 and 2018). The eGFR trend was also with the same pattern in participants with and without hypertension, independently in the sub-group analysis.

According to Table 4, the ALT trend of all participants was decreasing as time went by. The decrease, however, was not significant in the years 2016 and 2017 compared to 2015 (*p*-value = 0.88 and *p*-value = 0.16, respectively) and was only significant in 2018 compared to 2015, after adjusting for the other variables (*p*-value = 0.04).

According to Table 5, the overall AST in the whole-group analysis was increasing as time went by, after adjusting for the other variables. However, the increase was only significant in 2018 compared to 2015 (*p*-value = 0.02). In the years 2016 and 2017 compared to 2015 the increase was not significant (*p*-value = 0.47 and *p*-value = 0.45, respectively).

In determining whether the overall eGFR, ALT and AST trends were significant in the whole group and sub-analysis, we added the interaction term of [presence of hypertension*time] in the multivariable analysis. The overall trend of the eGFR was significant (*p*-value < 0.001) in the whole group analysis after adjusting for the other variables. For non-hypertensive and hypertensive participants, the independent overall trends were significant for both groups in the sub-group analysis (*p*-value < 0.001 and = 0.002 respectively). With regard to ALT, the overall trend was also significant (*p*-value 0.003). However, the overall trends in participants independently with and without hypertension was not significant in the sub-group analysis (*p*-value = 0.47 and *p*-value = 0.55, respectively). The AST overall trend of all participants throughout the course of the study was also significant in the whole group analysis (*p*-value < 0.001). The overall trend was also significant for hypertensive and non-hypertensive participants independently in the sub-group analysis (*p*-value < 0.001 and *p*-value = 0.001, respectively).

## 4. Discussion

The whole-group analysis of this study indicated that there was a significant association of hypertension with renal function, but not with liver function. Participants with hypertension had decreased eGFR compared to participants with no hypertension after adjustment of the other variables. Other factors that were found to have a significant association with eGFR included age, sex, ART duration and hypertension treatment. In the sub-group analysis, sex was only found to be significantly associated with eGFR in participants with hypertension, and the ART duration was only significantly associated with eGFR in participants with no hypertension. Factors with significant association with ALT in the whole-group analysis included age and sex. In the sub-group analysis, age did not have significant association with ALT in any of the two groups of participants. There were no factors that were found to have a significant association with AST in the whole-group analysis. In the sub-group analysis ART duration was found to have a significant association with AST in patients with no hypertension. The variations within the sub-group analysis and the whole-group analysis in all the independent variables in association with outcome variables can suggest a sub-group effect of having hypertension [18].

As participants with hypertension had decreased eGFR levels compared to participants without hypertension, such findings were in-line with those of studies done in Ethiopia [19], Brazil [20] and India [21]. The significant association of the presence of hypertension with renal function impairment can be explained by elevated levels of serum creatinine that are usually observed in patients with high blood pressure, compared to those with normal blood pressure [22]. It is also important to note that the kidney is usually the main aim of high blood pressure, and most kidney diseases are associated with such. On another note, the association of hypertension with eGFR can also be explained by that patients with hypertension and also taking ART are more likely to have kidney problems compared to non-hypertensive patients on ART, as some of anti-HIV drugs facilitate kidney dysfunction, and such process can be even much faster with presence of hypertension. Even the results of our study revealed that ART duration significantly contributes to eGFR.

With regard to sex, males were found to have increased eGFR than females after adjustment of the other variables. These results are in line with some reported in other studies [23,24,25]. There are different ideas or theories that can be used to explain this. During young adulthood, females might have increased eGFR than males, and such elevated eGFR might be concealed by scaling to body surface area, which may lead to eGFR declining at a faster rate [26]. Another explanation can be in relation to the differences in normal serum creatinine levels between males and females. The threshold for defining normal serum creatinine (which is very important when calculating eGFR) is different for males compared to females. Males usually have higher thresholds than females (110 µmol/L and 90 µmol/L, respectively) [27]. The significance of a single creatinine value is associated with a patient’s muscle mass [28,29], hence males tend to have higher serum creatinine levels compared to females [30]. It should be noted that for males to have higher levels of serum creatinine does not necessarily mean they are more prone to renal dysfunction than females. The higher levels are not health hazards but normal. The same thing can be said about the males’ eGFR.

Another factor that was found to have significant association with eGFR was age. From our results, eGFR was declining with age after adjusting for the other variables. Such results are consistent with those of other studies [24,31,32]. It is important to state that the categorization of renal impairment with age, more especially in the older population is a facet of continuing discourse and making conclusions about renal impairment in relation to age is beyond the scope of this study, especially when using eGFR.

Sex (with older age) was one of the factors found to have a significant association with ALT in the whole-group analysis. Males had increased ALT compared to females. This clearly explains why the threshold for defining normal ALT is different for males compared to females (35 U/L for males and 25 U/L for females) [14]. The higher levels of ALT associated with males should not be perceived as males being more at risk of liver diseases than females. They are just normal for males.

The retrospective cross-sectional study of the Fourth Korean National Health and Nutrition Examination Survey [14] recommended to lower the current ALT thresholds in order to identify individuals at risk for chronic liver disease. Schwimmer et al. forwarded a similar recommendation [33].

With regard to older age, as it has a significant association with ALT, more especially with the whole group analysis, lot of other studies have found similar association [34,35,36]. What is of great importance to state is that reduction in ALT levels does not necessarily mean there is a problem with the liver, but it can be an indication of other non-liver-related morbidity and mortality [35,37,38,39,40]. Similar studies have found lower ALT to be a biomarker for malnutrition, disability, sarcopenia and frailty. These kinds of calamities are very common with the older population, hence the lower ALT in the adult population should be interpreted in consideration of these calamities rather than liver problem alone. The potentially existing association between liver function impairment and lower ALT levels, more especially with the older population, needs more exploration.

In our study we found no significant association between hypertension and any of the liver function tests (LFTs). Previous studies examining the association of serum AST and ALT with hypertension reported inconsistent results [41,42,43]. To better understand the association of hypertension with AST and ALT, it is also important to understand the association of these LFTs with other NCDs where hypertension is a predisposing factor. For example, previous studies have reported a significant association between ALT and the development of diabetes, stroke, and cardiovascular disease [44,45,46]. That on its own is an illustration that the association of hypertension with liver enzymes needs further exploration, with good control of confounding factors.

With regard to eGFR, ALT and AST trends over the study time, we identified that they were significant for all participants. Indeed, changes are most likely to take place as time goes by in these lab tests for a number of reasons. For instance, studies have proven that the use Tenofovir-based therapy does have an effect on renal function [47,48,49], and some induce liver toxicity [50]. For such reasons we are most likely to see changes in eGFR, ALT/AST over time in HIV positive patients who are on ART. The changes can also be affected by the presence of hypertension, as increased blood volume and increased blood pressure increases GFR [51]. Hence in our study the difference in the changes of patients with hypertension compared to those with no hypertension were significant. Moreover, another reason that might lead to the significant changes in renal and liver function tests over time can be age. In our study we indeed find a significant association of older age with eGFR, ALT and AST. As time goes by patients becoming older, we can indeed see the changes in their liver and renal function tests, which can also be facilitated by ART regimes and presence of hypertension among other things. Therefore, it is of great importance to consistently monitor renal and liver function in patients on ART, more especially in the older patients and those with hypertension. As eGFR was found to be increasing over the study period in all participants, our results are in line with those found in other studies [52,53,54,55]. Such steady increase over time can be an indication of adequate treatment of an underlying nephropathy that was HIV related [47,56], and also adequate treatment of other acute ailments that might have been present during initiation of ART.

Even though it has been well established in many studies that some ART regimens have a significant association with renal and liver function, in our study we did not find such significance. That might likely be due to that part of our study methodology used to generate the data that did not have sufficient power to detect that dependence. However, we did find a significant association between LFTs, eGFR and ART duration. On a different note, it is also important to state that the insignificant association can be an indication that ART being developed recently has been tuned to have less side effects than the one of earlier days of ART establishment. Hence now studies have also found no significant association between tenofovir-based therapy and renal function are also existing [54,57,58,59].

We did not find a significant association between antihypertensive therapy and LFTs, but we did find a significant association between antihypertensive therapy and eGFR, more especially with the use of more than one drug class. The association was inversely proportional. In some articles about renal effects of antihypertensive drugs, the authors stated that acute deterioration of renal function may occur after administering beta-blockers, calcium channel blockers and ACE inhibitors [60,61]. Such deterioration might even be more severe when there is an underlying renal problem. These antihypertensive drugs facilitation on renal function deterioration might be because of the likelihood of the presence of bilateral renal artery stenosis, especially with ACE inhibitors [60]. This may explain the decrease in eGFR we observed in our study. Generally, in patients with normal renal function, antihypertensive therapy has been found to improve renal function in many studies and reviews [62,63,64,65,66,67,68]. Hence a conclusion has been made that well controlled blood pressure is one of the pre-requisites for proper kidney functioning [67]. Therefore, drugs that contribute to the control of blood pressure are believed to be renoprotective.

This study encountered several limitations. It was based on a retrospective analysis of already collected data from chronic patient files. Factors like alcohol use, BMI and smoking status were not available in the files or the necessary information needed to formulate some of such variables was missing. Moreover, there were variations in the initiation dates of the participants on ART, and hypertension diagnosis and treatment, making it hard to establish a causal relationship, even though this was a longitudinal study.

## 5. Conclusions

PLHIV on ART with hypertension had increased eGFR compared to those without hypertension. Hypertension did not have significant association with liver function tests (ALT and AST). That means conclusions on whether the presence of hypertension in PLHIV on ART aggravates the development of liver toxicity could not be made. More especially because drastic changes in these LFTs might not necessarily mean there is a problem with liver but can be an indication of other non-liver related morbidity and mortality. With regard to eGFR indicating a significant correlation between renal function, hypertension in PLHIV on ART, renal function of PLHIV with hypertension needs to be constantly monitored, more especially in older patients with hypertension, and those taking traditional β-blockers antihypertensive drugs. Factors that were found to have a significant association with eGFR in both groups of participants included age, sex, hypertension, ART duration, time of study, and hypertension treatment. Factors that were found to have a significant association with liver function tests (ALT and AST) included age, sex and time of study.

## Figures and Tables

**Table 1:** Demographic and Clinical Characteristics of Participants.

	Hypertension		Total (N = 525)	*p*-Value
	No (*n* = 303)	Yes (*n* = 222)		
Sex				
Female	181 (59.7%)	145 (65.3%)	326 (62.1%)	0.19
Male	122 (40.3%)	77 (34.7%)	199 (37.9%)	
Age				
30–40 years	164 (54.1%)	65 (29.3%)	229 (43.6%)	<0.001 *
41–50 years	99 (32.7%)	79 (35.6%)	178 (33.9%)	
>50 years	40 (13.2%)	78 (35.1%)	118 (22.5%)	
Mean Age ± SD	41.99 ± 7.144	47.34 ± 9.564	44.25 ± 8.660	
Settlement				
Rural	199 (65.7%)	134 (60.4%)	333 (63.4%)	0.21
Urban and Peri-Urban	104 (34.3%)	88 (39.6%)	192 (36.6%)	
Region				
Hhohho	32 (10.6%)	17 (7.7%)	49 (9.3%)	0.29
Manzini	203 (67.0%)	165 (74.3%)	368 (70.1%)	
Lubombo	50 (16.5%)	27 (12.2%)	77 (14.7%)	
Shiselweni	18 (5.9%)	13 (5.9%)	31 (5.9%)	
ART Regimen				
NVP-based	73 (24.1%)	98 (44.1%)	171 (32.6%)	<0.001 *
EFV-based	221 (72.9%)	123 (55.4%)	344 (65.5%)	
LPV/r-based	9 (3.0%)	1 (0.5%)	10 (1.9%)	
ART Duration				
≤5 years	149 (49.2%)	136 (61.3%)	285 (54.3%)	0.01 *
>5 years	154 (50.8%)	86 (38.7%)	240 (45.7%)	
Mean ART Duration ± SD	5.65 ± 1.523	5.37 ± 1.504	5.53 ± 1.520	
Had Adverse Effects				
No	297 (98.0%)	210 (94.6%)	507 (96.6%)	0.03 *
Yes	6 (2.0%)	12 (5.4%)	18 (3.4%)	
Adherence				
Poor	54 (17.8%)	36 (16.2%)	90 (17.1%)	0.69
Good	238 (78.5%)	175 (78.8%)	413 (78.7%)	
Excessive	11 (3.6%)	11 (5.0%)	22 (4.2%)	
W.H.O. HIV Staging				
Asymptomatic Stage	294 (97.0%)	203 (91.4%)	497 (94.7%)	0.01 *
Advanced Stages	9 (3.0%)	19 (8.6%)	28 (5.3%)	
Had Drug Substitution				
No	269 (88.8%)	194 (87.4%)	463 (88.2%)	0.63
Yes	34 (11.2%)	28 (12.6%)	62 (11.8%)	
Isoniazid Preventive Therapy				
No	144 (47.5%)	93 (41.9%)	237 (45.1%)	0.20
Yes	159 (52.5%)	129 (58.1%)	288 (54.9%)	
Hypertension Treatment				
None	303 (100%)	-	303 (57.7%)	<0.001 *
Lifestyle Modification	-	70 (31.5%)	70 (13.3%)	
Single Drug Class	-	80 (36.0%)	80 (15.2%)	
Two Drug Classes	-	55 (24.8%)	55 (10.5%)	
Three Drug Classes	-	17 (7.7%)	17 (3.2%)	

SD: standard deviation, N: total study population, *n*: subset of study population, *: statistically significant *p*-value, >: greater than, <: less than, INH: isoniazid, W.H.O.: World Health Organization, NVP: Nevirapine, EFV: Efavirenz, LVP/r:ss Lopinavir/Ritonavir, HIV: Human Immunodeficiency Virus, ART: Antiretroviral Therapy, -: irrelevant.

**Table 2:** ART Toxicity Related Outcomes.

	Hypertension				Total	
	No		Yes			
	*n*	Mean ± SD	*n*	Mean ± SD	N	Mean ± SD
Estimated Glomerular Filtration (eGFR)—mL/min						
2015	299	106.73 ± 23.24	214	96.64 ± 24.56	513	102.52 ± 24.29
2016	302	110.95 ± 25.98	221	99.12 ± 24.69	523	105.95 ± 26.09
2017	302	113.34 ± 20.95	221	101.96 ± 24.25	523	108.53 ± 23.08
2018	301	114.01 ± 22.06	216	102.68 ± 24.75	517	109.28 ± 23.86
Alanine Aminotransferase (ALT)—U/L						
2015	303	32.10 ± 32.01	220	31.32 ± 39.39	523	31.77 ± 35.27
2016	303	31.41 ± 25.80	222	28.00 ± 18.20	525	29.97 ± 22.94
2017	302	29.71 ± 19.12	222	28.01 ± 13.47	524	28.99 ± 16.96
2018	302	30.56 ± 22.11	219	30.82 ± 42.08	521	30.67 ± 32.02
Aspartate Aminotransferase (AST)—U/L						
2015	303	34.45 ± 24.26	220	32.63 ± 21.80	523	33.68 ± 23.25
2016	303	33.59 ± 18.71	222	29.96 ± 14.09	525	32.05 ± 16.99
2017	303	33.60 ± 14.56	222	31.66 ± 13.53	525	32.78 ± 14.15
2018	302	35.70 ± 18.90	219	34.96 ± 35.17	521	35.39 ± 26.94

N: total study population, *n*: subset of study population, SD: standard deviation.

**Table 3:** Factors Associated with eGFR.

	All Patients		Non-Hypertensive		Hypertensive	
	B (95% CI)	*p*-Value	B (95% CI)	*p*-Value	B (95% CI)	*p*-Value
Hypertension						
Yes	−2.22 (−6.21, 2.19)	0.03 *	-	-	-	-
No	Ref.		-		-	
Age						
>50 years	−20.29 (−24.37, −16.20)	<0.001 *	−21.94 (−27.19, −16.68)	<0.001 *	−19.12 (−25.36, −12.88)	<0.001 *
41–50 years	−8.96 (−12.64, −5.31)	<0.001 *	−8.47 (−13.18, −3.77)	<0.001 *	−8.82 (−14.80, −2.84)	0.004 *
30–40 years	Ref.		Ref.		Ref.	
Sex						
Male	3.94 (0.63, 7.25)	0.02 *	2.53 (−1.72, 6.78)	0.24	6.98 (1.60, 12.37)	0.01 *
Female	Ref.		Ref.		Ref.	
Settlement						
Urban and Peri-Urban	−1.27 (−4.70, 2.15)	0.47	−1.00 (−5.52, 3.51)	0.66	−0.84 (−6.26, 4.58)	0.76
Rural	Ref.		Ref.		Ref.	
ART Regimen						
LPV/r-based	−5.32 (−22.63, 12.00)	0.55	−12.04 (−32.24, 8.17)	0.24	9.32 (−3.37, 22.00)	0.15
EFV-based	3.10 (−0.22, 6.42)	0.07	3.07 (−1.75, 7.86)	0.21	2.85 (−2.06, 7.76)	0.26
NVP-based	Ref.		Ref.		Ref.	
Art Duration						
Year	−1.37 (−2.51, −0.24)	0.02 *	−1.60 (−3.13, −0.07)	0.04 *	−1.17 (−2.97, 0.63)	0.20
ART Adverse Effects						
Yes	−4.86 (−15.66, 5.95)	0.38	9.06 (−6.14, 24.25)	0.24	−10.98 (−24.55, 2.59)	0.11
No	Ref.		Ref.		Ref.	
W.H.O. HIV Stage						
Advanced Stage	1.45 (−5.25, 8.15)	0.67	−3.93 (−17.90, 10.05)	0.58	3.02 (−4.19, 10.23)	0.41
Asymptomatic Stage	Ref.		Ref.		Ref.	
Hypertension Treatment						
Three Drug Classes	−4.20 (−13.08, 4.69)	0.35	-	-	−5.17 (−14.43, 4.09)	0.27
Two Drugs Classes	−7.86 (−14.86, −0.85)	0.03 *	-	-	−7.53 (−14.68, −0.38)	0.04 *
Single Drug Class	−4.15 (−9.67, 1.37)	0.14	-	-	−4.09 (−9.52, 1.35)	0.14
Lifestyle Modification	Ref.		-		Ref.	
Study Duration						
2018	6.68 (4.75, 8.62)	<0.001 *	7.19 (4.88, 9.51)	<0.001 *	6.00 (2.72, 9.29)	<0.001 *
2017	5.97 (4.08, 7.87)	<0.001 *	6.47 (4.71, 8.76)	<0.001 *	5.273 (2.05, 8.50)	0.001 *
2016	3.40 (1.52, 5.27)	<0.001 *	4.01 (1.47, 6.54)	0.002 *	2.56 (−0.20, 5.33)	0.07
2015	Ref.		Ref.		Ref.	

*: statistically significant *p*-value, HIV: Human Immunodeficiency Virus, >: greater than, CI: confidence interval, <: less than, -: irrelevant, Ref.: reference.

**Table 4:** Factors Associated with ALT.

	All Patients		Non-Hypertensive		Hypertensive	
	B (95% CI)	*p*-Value	B (95% CI)	*p*-Value	B (95% CI)	*p*-Value
Hypertension						
Yes	2.55 (−2.64, 7.74)	0.34	-	-	-	-
No	Ref.					
Age						
>50 years	−2.10 (−4.33, 0.13)	0.07	−3.11 (−8.10, 1.87)	0.22	−2.29 (−8.67, 4.10)	0.48
41–50 years	−0.89 (−3.49, 1.72)	0.50	0.62 (−4.04, 5.28)	0.80	−0.33 (−7.10, 6.43)	0.92
30–40 years	Ref.		Ref.		Ref.	
Sex						
Male	3.44 (0.86, 6.03)	0.01 *	12.23 (7.68, 16.78)	<0.001 *	7.04 (1.74, 12.34)	0.01 *
Female	Ref.		Ref.		Ref.	
Settlement						
Urban and Peri-Urban	0.23 (−2.22, 2.68)	0.85	−1.15 (−4.97, 2.67)	0.56	0.83 (−4.85, 6.52)	0.77
Rural	Ref.		Ref.		Ref.	
ART Regimen						
LPV/r-based	−6.88 (−17.34, 3.58)	0.20	−8.66 (−19.94, 2.61)	0.13	−10.78 (−23.78, 2.21)	0.10
EFV-based	−0.41 (−2.88, 2.07)	0.75	3.96 (−1.41, 9.33)	0.15	0.30 (−4.52, 5.13)	0.90
NVP-based	Ref.		Ref.		Ref.	
Art Duration						
Year	0.34 (−0.29, 0.98)	0.29	0.23 (−1.19, 1.66)	0.75	0.15 (−1.61, 1.91)	0.87
ART Adverse Effects						
Yes	0.32 (−5.13, 5.77)	0.91	−0.30 (−11.24, 10.64)	0.96	−5.07 (−10.81, 0.68)	0.08
No	Ref.		Ref.		Ref.	
W.H.O. HIV Stage						
Advanced Stage	−0.35 (−3.88, 3.18)	0.85	−5.32 (−13.16, 2.52)	0.18	−2.55 (−7.80, 2.70)	0.34
Asymptomatic Stage	Ref.		Ref.		Ref.	
Hypertension Treatment						
Three Drug Classes	−2.74 (−9.53, 4.05)	0.43	-	-	5.19 (−1.51, 11.89)	0.13
Two Drugs Classes	−1.48 (−6.70, 3.74)	0.58	-	-	−2.95 (−9.04, 3.14)	0.34
Single Drug Class	−2.25 (−7.25, 2.76)	0.38	-	-	−1.082	0.73
Lifestyle Modification	Ref.		-		Ref.	
Study Duration						
2018	−2.80 (−5.51, −0.09)	0.04 *	−1.45 (−5.33, 2.43)	0.47	−0.70 (−8.65, 7.24)	0.86
2017	−1.79 (−4.32, 0.73)	0.16	−2.12 (−5.74, 1.51)	0.25	−3.49 (−8.96, 1.98)	0.21
2016	−0.19 (−2.66, 2.28)	0.88	−0.59 (−4.03, 2.86)	0.74	−3.44 (−9.02, 2.15)	0.23
2015	Ref.		Ref.		Ref.	

*: statistically significant *p*-value, HIV: Human Immunodeficiency Virus, >: greater than, CI: confidence interval, <: less than, -: irrelevant, Ref.: reference.

**Table 5:** Factors Associated with AST.

	All Patients		Non-Hypertensive		Hypertensive	
	B (95% CI)	*p*-Value	B (95% CI)	*p*-Value	B (95% CI)	*p*-Value
Hypertension						
Yes	−2.11 (−5.31, 1.09)	0.20	-	-	-	-
No	Ref.		-		-	
Age						
>50 years	0.67 (−1.30, 2.64)	0.50	2.00 (−0.75, 4.74)	0.15	−0.76 (−3.76, 2.24)	0.62
41–50 years	0.95 (−1.12, 3.01)	0.37	1.97 (−0.40, 4.35)	0.10	−0.84 (−4.54, 2.85)	0.66
30–40 years	Ref.		Ref.		Ref.	
Sex						
Male	0.84 (−1.49, 3.18)	0.48	1.73 (−0.33, 3.79)	0.10	−0.61 (−3.81, 2.59)	0.71
Female	Ref.		Ref.		Ref.	
Settlement						
Urban and Peri-Urban	−0.38 (−2.29, 1.53)	0.70	1.23 (−0.97, 3.43)	0.27	−1.74 (−4.65, 1.17)	0.24
Rural	Ref.		Ref.		Ref.	
ART Regimen						
LPV/r-based	3.58 (−7.85, 15.02)	0.54	7.04 (−7.77, 21.85)	0.35	−3.31 (−15.29, 8.67)	0.59
EFV-based	1.21 (−0.60, 3.02)	0.19	1.42 (−1.34, 4.17)	0.32	0.26 (−2.40, 2.92)	0.85
NVP-based	Ref.		Ref.		Ref.	
ART Duration						
Year	−0.32 (−0.87, 0.25)	0.25	−1.13 (−1.92, −0.35)	0.01 *	0.53 (−0.31, 1.36)	0.22
ART Adverse Effects						
Yes	−2.15 (−6.41, 2.10)	0.32	−6.95 (−16.98, 3.08)	0.18	1.01 (−3.67, 5.70)	0.67
No	Ref.		Ref.		Ref.	
W.H.O. HIV Stage						
Advanced Stage	−0.74 (−4.19, 2.72)	0.68	2.55 (−2.82, 7.91)	0.35	−1.34 (−5.86, 3.18)	0.56
Asymptomatic Stage	Ref.		Ref.		Ref.	
Hypertension Treatment						
Three Drug Classes	5.17 (−1.40, 11.73)	0.12	-	-	4.85 (−1.52, 11.21)	0.14
Two Drugs Classes	0.09 (−2.99, 3.16)	0.96	-	-	0.53 (−2.36, 3.42)	0.72
Single Drug Class	1.82 (−1.86, 5.50)	0.33	-	-	1.50 (−2.04, 5.03)	0.41
Lifestyle Modification	Ref.		-		Ref.	
Study Duration						
2018	2.31 (0.39, 4.22)	0.02 *	2.21 (0.43, 3.98)	0.02 *	2.49 (−1.40, 6.37)	0.21
2017	0.62 (−0.99, 2.21)	0.45	0.47 (−1.20, 2.13)	0.58	0.83 (−2.18, 3.85)	0.59
2016	−0.58 (−2.15, 0.99)	0.47	−0.44 (−2.03, 1.15)	0.59	−0.80 (−3.62, 2.03)	0.58
2015	Ref.		Ref.		Ref.	

*: statistically significant *p*-value, HIV: Human Immunodeficiency Virus, >: greater than, CI: confidence interval, <: less than, -: irrelevant, Ref.: reference.

## Data Availability

The data sets used and analyzed during the current study are available from the corresponding author on reasonable request.

## Abbreviations

ARVs	Antiretrovirals
CDC	U.S. Center for Disease Control and Prevention
CKD	Chronic Kidney Disease
CYPs	Cytochromes P450
DBP	Diastolic Blood Pressure
EsMoH	Eswatini Ministry of Health
HAART	Highly Active Antiretroviral Therapy
INH	Isonicotinylhydrazide (Isoniazid)
IQR	Interquartile Range
LFTs	Liver Function Tests
MDRD	Modification of Diet in Renal Disease
NICE	National Institute for Health and Care Excellence
NNRTIs	Non-nucleoside Reverse Transcriptase Inhibitors
NRTIs	Nucleoside Reverse Transcriptase Inhibitors
PAGAA	Panel on Antiretroviral Guidelines for Adults and Adolescents
PAHO	Pan American Health Organization
PEPFAR	President’s Emergency Plan for AIDS Relief
PIs	Protease Inhibitors
PLHIV	People Living with HIV
RFMH	Raleigh Fitkin Memorial Hospital
SBP	Systolic Blood Pressure
SHIMS	Swaziland HIV Incidence Measurement Survey
SNAP	Eswatini National Program
USAID	United States Agency for International Development
USDHHS	United States Department of Health of Human Services
